# Efficacy of K-wire tension band fixation compared with other alternatives for patella fractures: a meta-analysis

**DOI:** 10.1186/s13018-018-0919-6

**Published:** 2018-09-05

**Authors:** Yinwang Zhang, Zhen Xu, Wuxue Zhong, Fuhai Liu, Jie Tang

**Affiliations:** Department of Orthopedics, Shanghai Xuhui District Central Hospital, No.966, Middle Huaihai Road, Shanghai, 200031 China

**Keywords:** K-wire tension band fixation, Patella fractures, Cannulated screws, Cable pin, Ring pin, Meta-analysis

## Abstract

**Background:**

To compare the efficacy and safety of K-wire tension band fixation (KTB) with other alternative approaches (cannulated screws, cable pin, and ring pin) for treatment of patella fractures by performing a meta-analysis.

**Methods:**

PubMed and EMBASE databases were searched for all relevant studies. Standardized mean difference (SMD) or relative risk (RR) and their corresponding 95% confidence intervals (CIs) were calculated for continuous or dichotomous outcomes via either a fixed- or random-effect model using Stata 13.0 software.

**Results:**

Nine literatures involving 949 patients (581 in the KTB group and 368 in the control group) were included. Pooled analysis showed there were no differences in the success rate, operative time, healing time, and number of infections between patients undergoing KTB and others. However, the incidence of complications (RR = 8.04, 95% CI = 4.45–14.53; *p* < 0.001) and VAS (SMD = 0.642, 95% CI = 0.22–1.06; *p* = 0.003) were lower, while flexion degree (SMD = − 0.70 95% CI = − 1.04–− 0.36; *p* < 0.001), Böstman joint function score (SMD = − 0.68, 95% CI = − 1.10–− 0.27; *p* = 0.001), Iowa knee score (RR = 0.88, 95% CI = 0.81–0.96; *p* = 0.004), and Lysholm score (SMD = − 0.71, 95% CI = − 1.10–− 0.32; *p* < 0.001) were significantly higher in patients undergoing alternative approaches than the KTB. Subgroup analysis also demonstrated the cannulated screw fixation was superior to KTB in reducing the incidence of complications.

**Conclusions:**

Alternative treatments may be effective for management of patella fractures and should be attempted to be popularized in clinic.

## Introduction

Patellar fractures have been a common clinical injury because of frequent traffic accidents and industrial accidents recently, with an estimated incidence of 13.1/100,000 per year, especially predominant in patients aged 20 and 50 years [1]. The main function of patella is to increase the force of quadriceps apparatus by improving the leverage and then maintain the extension of knee joint. Furthermore, the intact patella protects the anterior articular surface of distal femur against external violence [2]. Hereby, fractures in the patella may lead to extension strength weakness, limited range of motion (ROM) of the knee joint, and patellofemoral or tibiofemoral arthritis, which all seriously influence the health-related quality of life of patients [3]. Therefore, how to manage patellar fractures to restore the functions of the patella has been a challenge for orthopedic surgeons.

Currently, the most commonly used surgical intervention for treatment of patellar fractures is open reduction and internal fixation with Kirschner wire (K-wire) tension band (KTB; modified or not) [2, 4–7]. This technique has been reported to provide satisfactory reduction outcomes in 90% of patients by converting the tension forces acting on the anterior surface into compression forces at the articular surface [6]. However, some studies indicate the incidence of postoperative complications, including wire breakage, migration and subsequently induced skin irritation, infection, pain, and reduction loss, may be high (approximately 21–53%) [8, 9]. In addition, the long-term function improvement for the knee joint may be also limited [10]. To overcome these disadvantages of KTB, several alternative approaches have been introduced, including closed reduction and fracture fixation using cannulated screws or inter-fragmentary screws (cable pin) with or without supplementary tension band wiring through the screw [4, 5, 8, 10–14] or open fixation with ring pin [15, 16]. Theoretically, the screws provide stronger fixation strength than Kirschner wires according to the biomechanical testing [17] and thus can protect the implants from breakage, migration, and related complications to improve the reduction and function outcomes. However, the comparative study results seemed to be controversial. For example, Tian et al. found that the Iowa knee score was significantly improved by the cannulated lag screw technique compared with KTB, with the excellent and good rate of 100% (49/49) and 86.5 (45/52), respectively [8]. But, the study of Wang et al. found that there was no significant difference in the Iowa knee score between two groups [9]. Flexion degree was considered to be significantly improved at 24-month follow-up in the study of Mao et al. [10]. Nevertheless, Lin et al. found that the superiority of improvement in the flexion degree was not present after a 6-month follow-up [5]. The pain relief effects were also differential among different studies [4, 5, 10]. This discrepancy may be attributed to the small sample size. Thus, it is essential to further confirm whether the above alternative approaches provide more excellent effects than KTB for patellar fractures.

The goal of this study was to comprehensively determine the efficacy of KTB by performing a meta-analysis of all controlled trials comparing KTB with all other alternative treatments for patellar fractures.

## Materials and methods

### Search strategy

This meta-analysis was performed in accordance with the Preferred Reporting Items for Systematic Reviews and Meta-Analyses (PRISMA) statement [18].

PubMed and EMBASE databases were systematically searched to screen all relevant studies published until January 2018. The search terms used included (patella fractures) AND (cable pin) OR (cannulated screw) OR (tension band wire). Furthermore, additional potentially relevant articles were also screened manually by reviewing the reference lists of retrieved articles.

### Selection criteria

Articles eligible in this meta-analysis had to meet the following inclusion criteria: (1) patients diagnosed with patella fractures; (2) patients receiving Kirschner tension band wire treatment; (3) clinical study comparing Kirschner tension band wire with a control; (4) relatively complete research data; and (5) only English publication languages. Studies were excluded if they met the following criteria: (1) did not have a control, such as case or cohort studies; (2) did not evaluate clinical results, such as animal studies; (3) the publications were abstracts, reviews, editorials, corresponding letters, or comments; (4) non-English publication; and (5) studies not providing inadequate data.

### Data extraction and quality assessment

Two reviewers independently screened eligible studies from the databases and extracted the following data: general characteristics (the first author, publication year, and region of study origin), research design (interventions and follow-up), patients (number, sex, age, injury reasons, and preoperative delay), and therapeutic outcomes [such as success rate, operative time, fracture healing time, the incidence of postoperative complications, the number of infections, flexion degree, pain (VAS, visual analogue score), the Böstman joint function score, Lysholm score, and Iowa knee score]. Any discrepancy was resolved through discussion or consultation with a third reviewer during study screening and data abstraction.

The quality of included study was evaluated by using a 7-point modified “Jadad” scoring system that assessed randomization, double-blinding, allocated concealment, participant withdrawals or dropouts, and intent to treat (ITT) [19]. Studies were considered to be of high quality if the Jadad score was ≥ 4.

### Statistical analysis

Heterogeneity between the trials was tested by using Chi-square and *I*^2^ statistics tests. *p* < 0.1 and *I*^2^ > 50% were used to indicate a significant heterogeneity between studies, and then a random-effects model was used to pool the study results; otherwise, a fixed-effects model was adopted. A standardized mean difference (SMD; for continuous variables) or the relative risk (RR; for dichotomous variables) and their corresponding 95% confidence intervals (CIs) were estimated as a measure of effect size. Egger’s test was used to assess the possible publication bias for continuous variables, while Harbord’s weighted linear regression test was applied for dichotomous outcomes [20, 21] *p* < 0.05 was considered to be statistically significant. Meta-analysis was conducted by Stata 13.0 software (STATA Corporation, College Station, TX, USA).

## Results

### Description of studies

**Fig. 1:**
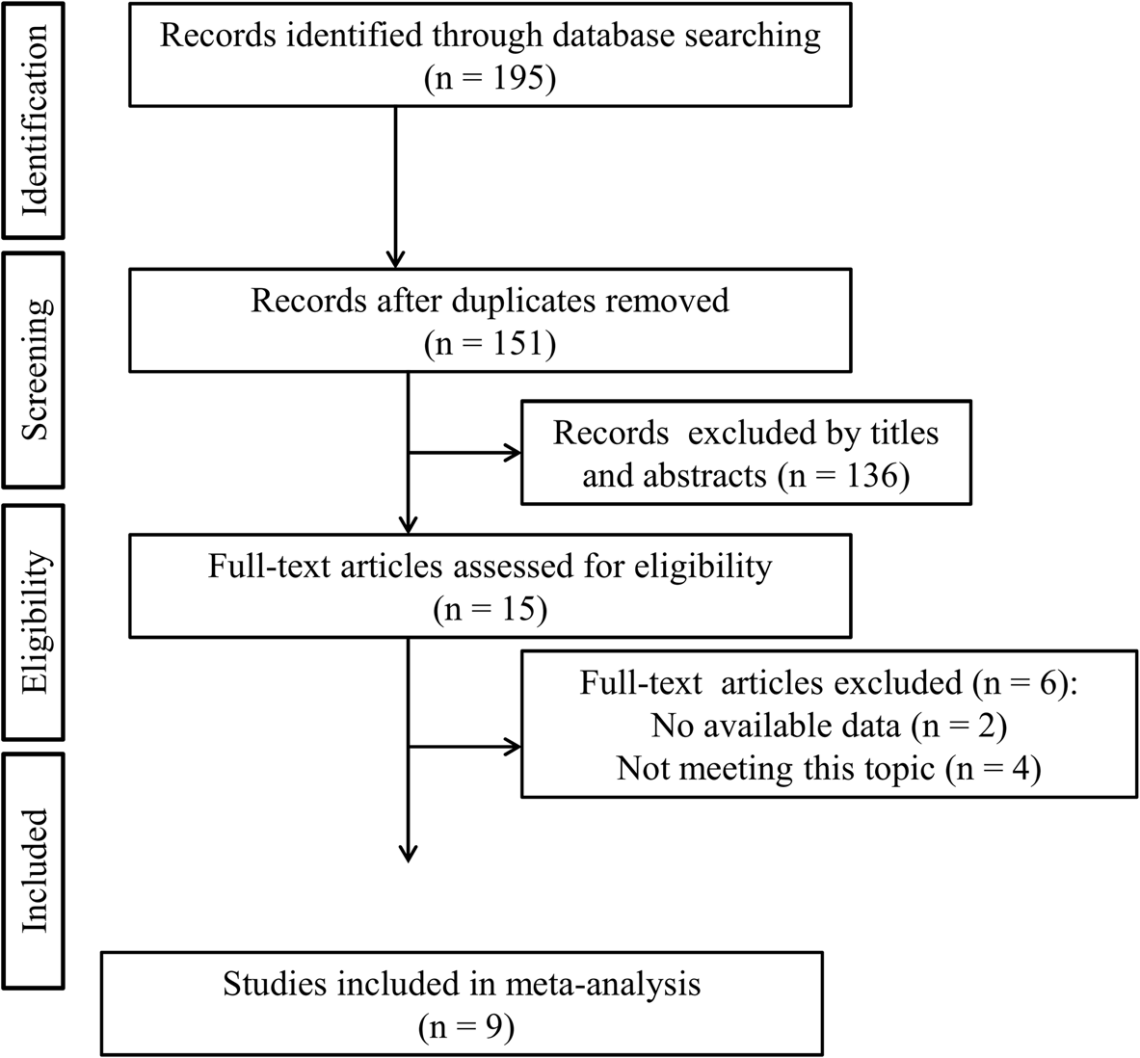
Flow diagram of literature screening process

The flow diagram of the literature search is shown in Fig. 1. Nine control studies with a total of 949 patients (581 in the KTB group and 368 in the control group) were ultimately considered to be eligible according to the inclusion and exclusion criteria [4, 5, 8–13, 15]. The characteristics of the included studies are presented in Table 1. Five studies compared KTB with cannulated screw tension band [4, 5, 8, 9, 11, 13], two studies compared with cable pin system [10, 12], and one compared with ring pin tension band [15]. Six studies were performed in China [4, 5, 8–10, 12], one in Taiwan [13], one in the USA [11], and the other one in Korea [15]. The patients in the included studies were middle-aged and elderly (an average age range, 40.2 to 60.2 years) and most of them were female (59.7%, 567/949). The last follow-up duration was longer than 12 months. According to the modified Jadad score, all the included trials were of high quality (Table 2).

**Table 1:** Characteristics of the 9 studies included in the meta-analysis

Reference		No.	Mean age	Female (%)	Injury reason	Preoperative delay (d)	Follow-up time (m)	Treatment method
Tian Y et al. 2011 [8]	China	52/49	56.12 ± 16.64 vs 57.12 ± 15.00	23 (44.2%) vs 30(61.2%)	Fall and slip, car accident	Unclear	12–36	MKTB* vs titanium cable-cannulated screw tension band
Mao N et al. 2013 [10]	China	20/20	43.5 ± 11.4 vs 40.2 ± 10.0	9 (22.5%) vs 6(15%)	Fall, car accident, sport injury	1.41 ± 0.31 vs 1.28 ± 0.41	24	KTB^#^ vs cable pin system
Hoshino CM et al. 2013 [11]	USA	315/133	60 (48–71) vs 58(43–68)	222 (70.5%) vs 92(69.2%)	Unclear	Unclear	34(19–52) vs 30(20–52)	KTB vs cannulated screw tension band
Wang CX et al. 2014 [9]	China	37/35	56.12 ± 16.64 vs 57.12 ± 15.00	18 (48.6%) vs 16(45.7%)	Fall, car accident,	Unclear	12–36	MKTB vs titanium cable-cannulated screw tension band
Lin T et al. 2015 [5]	China	26/26	52.5 ± 17.4 vs 50.8 ± 16.3	13 (50.0%) vs 13(42.3%)	Fall, sport injury, car accident,	3.3 ± 1.4 vs 2.8 ± 1.2	12	MKTB vs cannulated screw tension band
Tan H et al. 2016 [4]	China	29/26	43.5 ± 11.4 vs 40.2 ± 10.0	7 (24.1%) vs 7(26.9%)	Fall, car accident, sport injury	Unclear	20.79 ± 5.36 vs 21.89 ± 4.72	MKTB vs cannulated screw tension band
Kyung MG et al. 2017 [15]	Korea	23/25	55 (24–83) vs 57(31–82)	15 (65.2%) vs 14(60.9%)	Unclear	Unclear	> 12	KTB vs ring pin tension band
Tian QX et al. 2015 [12]	China	39/34	44.5 ± 12.8 vs 46.2 ± 14.2	25 (64.1%) vs 21(61.8%)	Unclear	3.1 ± 2.4 vs 3.5 ± 2.3	19.10 ± 9.31 vs 18.56 ± 8.67	KTB vs cable pin system
Chiang CC et al. 2011 [13]	Taiwan	40/20	60.2 ± 15.4 vs 56.6 ± 14.7	25 (62.0%) vs 11(55.0%)	Fall, traffic accident	0.7 ± 0.5 vs 0.6 ± 0.3	36.6 ± 7.4 vs 38.3 ± 6.8	KTB vs cannulated screw tension band

*MKTB Modified K-wire tension band; ^#^*KTB* K-wire tension band

**Table 2:** Methodologic quality of included studies

Reference	No.^a^	Randomization	Blinding	Allocated concealment	Baseline data	Follow-up	Withdraw lost to follow-up	ITT^b^	Quality level
Tian Y et al. 2011 [8]	101	Not used	Not used	Unclear	Comparable	Yes	Described	Yes	4.5/7
Mao N et al. 2013 [10]	40	Yes	Not used	Adequate	Comparable	Yes	Described	Yes	6/7
Hoshino CM et al. 2013 [11]	448	Not used	Not used	Unclear	Comparable	Yes	Described	Yes	5.5/7
Wang CX et al. 2014 [9]	72	Not used	Not used	Unclear	Comparable	Yes	Described	Yes	5/7
Lin T et al. 2015 [5]	52	Yes	Not used	Adequate	Comparable	Yes	Described	Yes	6/7
Tan H et al. 2016 [4]	55	Not used	Not used	Adequate	Comparable	Yes	Described	Yes	5/7
Kyung MG et al. 2017 [15]	48	Not used	Not used	Unclear	Comparable	Yes	Described	Yes	4.5/7
Tian QX et al. 2015 [12]	73	Yes	Not used	Adequate	Comparable	Yes	Described	Yes	6/7
Chiang CC et al. 2011 [13]	60	Not used	Not used	Adequate	Comparable	Yes	Described	Yes	5/7

^a^Sample size

^b^Intention-to-treat

### Main outcomes

Success rate was evaluated in four studies. Obvious heterogeneity was found across these four trials (*p* < 0.001, *I*^2^ = 90.5%), and thus a random-effects model was performed. The pooled results suggested that there was no difference in the success rate (RR = 0.82, 95% CI = 0.67–1.01; *p* = 0.056) between patients undergoing KTB and others.

Operative time was assessed in five studies. Evidence heterogeneity was present across these five trials (*p* < 0.001, *I*^2^ = 85.5%), and thus a random-effects model was performed. The combined results implied that there was no difference in the operative time between KTB and other treatments (SMD = 0.23, 95% CI = − 0.40–0.85; *p* = 0.476).

Six trials were included in the meta-analysis to evaluate the fracture healing time. Significant heterogeneity was detected across these six trials (*p* < 0.001, *I*^2^ = 69.6%), and thus a random-effects model was adopted. The pooled analysis showed that the fracture healing time after KTB was not longer than other treatments (SMD = 0.32, 95%CI = − 0.04–0.68; *p* = 0.085).

**Fig. 2:**
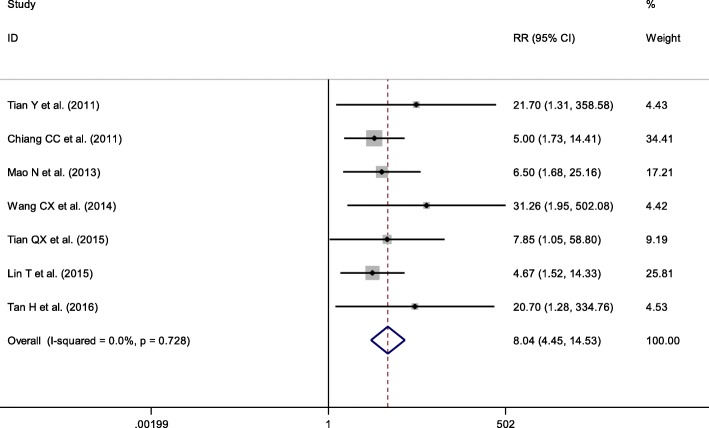
Forest plots for the incidence of complications between KTB and other treatments. CIs, confidence intervals; RR, relative risk

Seven trials were included in the meta-analysis to evaluate the incidence of complications (such as displaced fragment, painful hardware, or implant migration which required implant removal or reoperation). No heterogeneity was present across these seven trials (*p* = 0.728, *I*^2^ = 0%), and thus a fixed-effects model was used. The combined results indicated that the complication risk after KTB may be higher than other treatments (RR = 8.04, 95% CI = 4.45–14.53; *p* < 0.001) (Fig. 2).

Four trials were included in the meta-analysis to evaluate the incidence of infection. No heterogeneity was observed across these four trials (*p* = 0.953, *I*^2^ = 0%), and thus a fixed-effects model was used. The combined results indicated that the incidence of complications was not statistically different between KTB and other treatments (RR = 2.82, 95% CI = 0.96–8.25; *p* = 0.058).

**Fig. 3:**
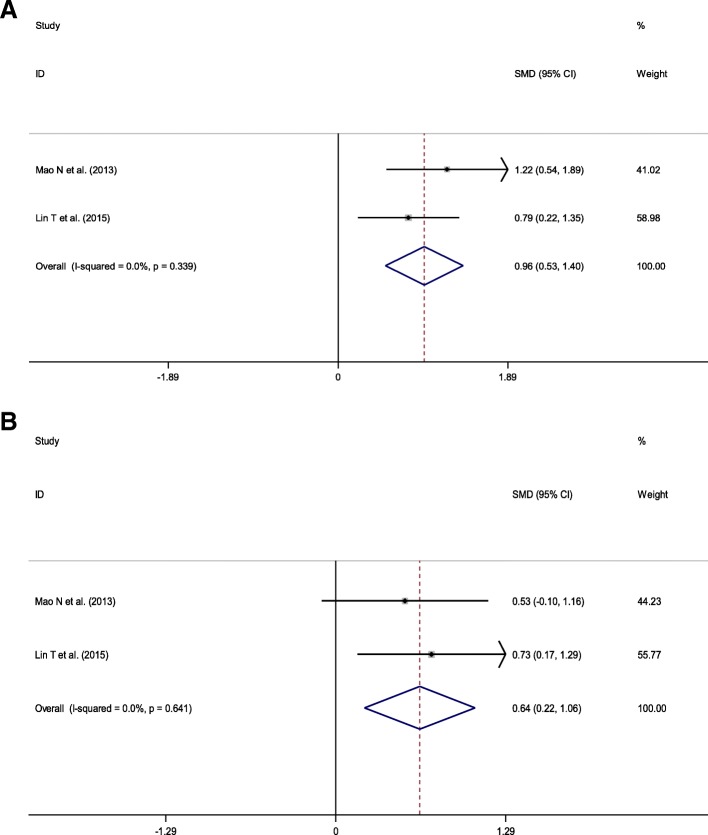
Forest plots for the VAS outcomes between KTB and other treatments. **a** 3-month; **b** 6-month. SMD, standardized mean difference; CIs, confidence intervals; RR, relative risk

Two trials were included in the meta-analysis to evaluate the pain. No heterogeneity was observed across these two trials (3-month: *p* = 0.339, *I*^2^ = 0%; 6-month: *p* = 0.641, *I*^2^ = 0%), and thus a fixed-effects model was used. The combined results indicated that the VAS score was significantly higher (worse) in the open KTB group than that in the group undergoing other treatments at 3-month (SMD = 0.96, 95% CI = 0.53–1.40; *p* < 0.001) (Fig. 3a) and 6-month follow-up (SMD = 0.642, 95% CI = 0.22–1.06; *p* = 0.003) (Fig. 3b).

**Fig. 4:**
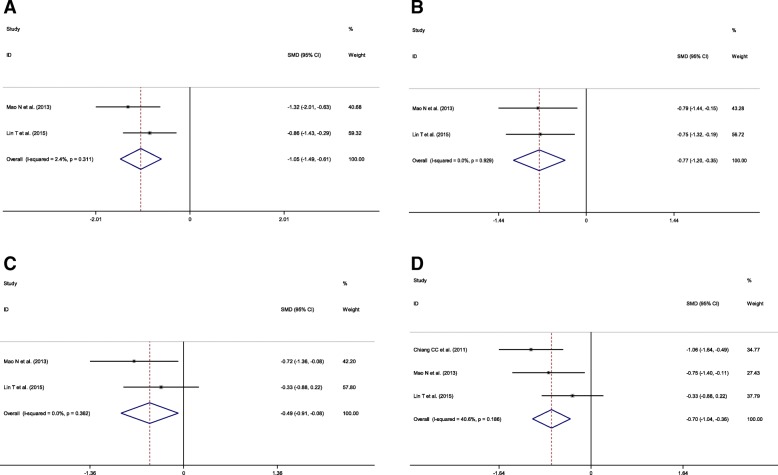
Forest plots for the flexion degree outcomes between KTB and other treatments. **a** 3-month; **b** 6-month; **c** 12-month; **d** last follow-up. SMD, standardized mean difference; CIs, confidence intervals; RR, relative risk

Two trials were included in the meta-analysis to evaluate the flexion degree at 3- , 6- , and 12-month follow-up. No heterogeneity was observed across these two trials (3-month: *p* = 0.311, *I*^2^ = 2.4%; 6-month: *p* = 0.929, *I*^2^ = 0%; 12-month: *p* = 0.362, *I*^2^ = 0%), and thus a fixed-effects model was used. The combined results indicated that the other treatment groups had gained more extension degree at 3- (SMD = − 1.05, 95% CI = − 1.49–− 0.61; *p* = 0.000) (Fig. 4a), 6- (SMD = − 0.77, 95% CI = − 1.20–− 0.35; *p* = 0.000) (Fig. 4b), and 12-month (SMD = − 0.49, 95% CI = − 0.91–− 0.08; *p* = 0.020) follow-up (Fig. 4c). Three trials were included to evaluate the flexion degree at the last follow-up time. No heterogeneity was also observed for these three studies (*p* = 0.186, *I*^2^ = 40.6%). The fixed-effects model analysis indicated that the other treatment groups had gained more extension degree at the last follow-up time (SMD = − 0.70 95%CI = − 1.04–− 0.36; *p* < 0.001) (Fig. 4d).

**Fig. 5:**
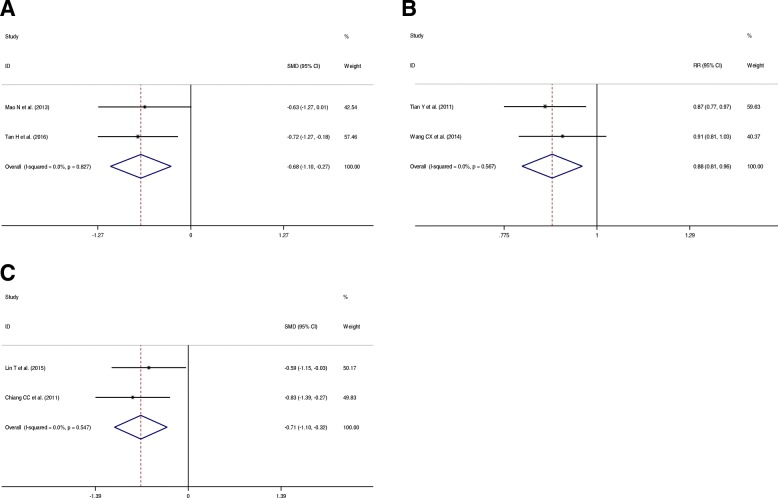
Forest plots for the function outcomes between KTB and other treatments. **a** Böstman score; **b** Iowa knee score; **c** Lysholm score. SMD, standardized mean difference; CIs, confidence intervals; RR, relative risk

Two trials were included in the meta-analysis to evaluate the Böstman joint function score. No heterogeneity was observed across these two trials (*p* = 0.827, *I*^2^ = 0%), and thus a fixed-effects model was used. The combined results indicated that the Böstman score was significantly lower in the open KTB group at the last follow-up time (SMD = − 0.68, 95% CI = − 1.10–− 0.27; *p* = 0.001) (Fig. 5a).

Two trials were included in the meta-analysis to evaluate the Iowa knee score. No heterogeneity was present across these two trials (*p* = 0.567, *I*^2^ = 0%), and thus a fixed-effects model was used. The combined results indicated that the excellent and good rate was significantly lower in the open KTB group at the last follow-up time (RR = 0.88, 95% CI = 0.81–0.96; *p* = 0.004) (Fig. 5b).

Two trials were included in the meta-analysis to evaluate the Lysholm score. No heterogeneity was observed across these two trials (*p* = 0.547, *I*^2^ = 0%), and thus a fixed-effects model was used. The combined results indicated that the Lysholm score was significantly lower in the open KTB group at the last follow-up time (SMD = − 0.71, 95% CI = − 1.10–− 0.32; *p* < 0.001) (Fig. 5c).

### Subgroup analysis

Subgroup analysis according to the comparison of KTB with cannulated screw tension band was also attempted. Due to the limited studies included, the meta-analysis only could be performed for the success rate, operative time, the fracture healing time, the incidence of infection, the incidence of complications, and flexion degree at the last follow-up. The pooled results indicated other treatments were superior to KTB in reducing the incidence of complications (RR = 8.43, 95% CI = 4.22–16.85; *p* < 0.001), but no significant differences in the operative time (SMD = 0.40, 95% CI = − 0.46–1.26; *p* = 0.360), fracture healing time (SMD = 0.19, 95%CI = − 0.03–0.40; *p* = 0.093), incidence of infection (RR = 2.98, 95% CI = 0.89–9.93; *p* = 0.076), and flexion degree (SMD = − 0.69, 95%CI = − 1.41–0.03; *p* = 0.06) were detected between two groups. The result for the success rate was similar to the overall outcomes.

### Publication bias

**Fig. 6:**
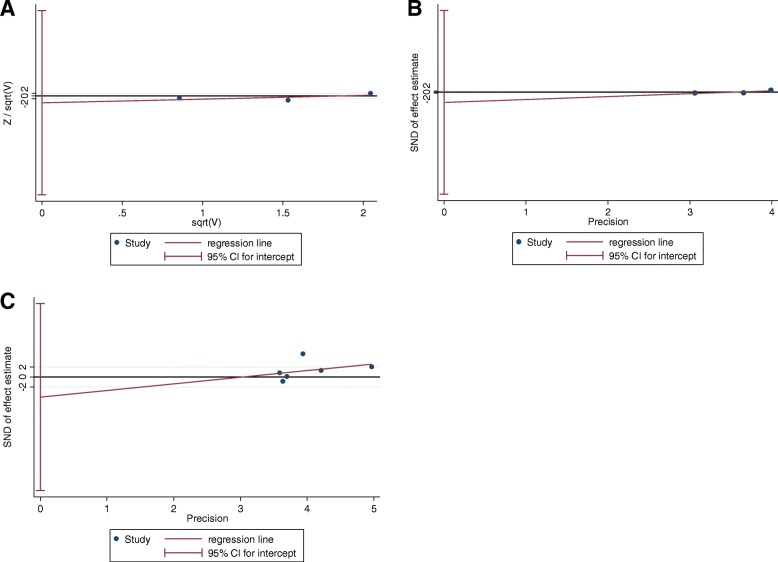
Publication bias. **a** the success rate; **b** the operative time; **c** the fracture healing time

Harbord’s weighted linear regression or egger test indicated no significant publication bias in the success rate (*p* = 0.521; Fig. 6a), operative time (*p* = 0.387; Fig. 6b), and fracture healing time (*p* = 0.583; Fig. 6c), where heterogeneity was observed in the above meta-analysis.

## Discussion

In present study, we, for the first time, used a meta-analysis to comprehensively compare the treatment effects of KTB with other strategies for patella fractures. Pooled results indicated alternative treatment strategies reduced the incidence of complications, VAS score and increased flexion degree, the Böstman joint function score, Iowa knee score, and Lysholm score in the short and long-term follow-up compared with KTB, indicating these alternative operative methods (cannulated screws, cable pin, and ring pin) may be more effective and safe for management of patella fractures, which was consistent with previous studies [12, 13, 22]. The superiority of alternative operations may be attributed to the following reasons: (1) compared with open reduction and internal fixation with KTB, closed reduction and fracture fixation using cannulated screws, or inter-fragmentary screws (cable pin) is minimally invasive. Smaller incision and less soft tissue dissection allows early mobilization and faster recovery, leading to improved function outcomes; (2) previous biomechanical comparisons have revealed that the screw fixation system may provide more stable and rigid fixation than the tension band wiring, showing significant lower displacement of the fracture gap during polycyclic loading [23] and higher resistance against the distraction forces [24]. The cable pin system could contact the bone surface more tightly and provide more compression [10]. The ring pin locked the implant to the patella, which also improved the fixation stability [15]. These may ultimately prevent the occurrence of implants loosening and reduction loss.

There are several limitations of our meta-analysis that should be taken into account when interpreting the results of our meta-analysis. First, several included studies were retrospectively performed, and patients were not randomly assigned to receive different operations, which may introduce unavoidable bias. Second, most of the studies assessed the short-term effectiveness (within 6 months), and the follow-up time was different among studies. Thirdly, the articles included in this meta-analysis were limited to those published up to January 2018; thus, some relevant unpublished studies may be missed. Fourthly, the sample size of included studies was relatively small, which led to the unavailable statistics for some postoperative outcomes (i.e., ROM). Fifthly, only the differences between KTB and modified metallic fixation methods (cannulated screws, cable pin, or ring pin) were compared in present study due to the limitation of inclusion and exclusion criteria. The studies of non-metallic substitutes for KTB [22, 25] were not involved. Accordingly, further comprehensive meta-analysis studies with prospective, randomized designs are still needed to confirm the superiority of KTB alternatives, including metallic and non-metallic implants.

## Conclusion

Our findings suggest that alternative treatment strategies may be more effective for management of patella fractures than KTB in reducing the incidence of complications, VAS score and increasing flexion degree, the Böstman joint function score, Iowa knee score, and Lysholm score and thus should be recommended in clinic.

## Abbreviations

CIs: Confidence intervals
ITT: Intent to treat
KTB: Kirschner wire tension band
PRISMA: Preferred Reporting Items for Systematic Reviews and Meta-Analyses
ROM: Range of motion
RR: Relative risk
SMD: Standardized mean difference
VAS: Visual analogue score
